# Hyperhaploid karyotypes in multiple myeloma

**DOI:** 10.18632/oncotarget.20875

**Published:** 2017-09-14

**Authors:** Jeffrey R. Sawyer, Gareth J. Morgan

**Affiliations:** Jeffrey R. Sawyer: Department of Pathology, Myeloma Institute, University of Arkansas for Medical Sciences, Little Rock, AR, USA

**Keywords:** hyperhaploidy, multiple myeloma, cytogenetics

Chromosome aneuploidies generate genetic diversity and subclonal heterogeneity in the progression of human cancers. In multiple myeloma (MM), there is consensus that chromosomal aneuploidies involve two primary numerical subgroups with different prognostic significance [1]. The largest numerical subgroup is comprised of hyperdiploid karyotypes characterized by the gain of a set of odd-numbered chromosomes including trisomies for chromosomes 3, 5, 7, 9, 11, 15, 19, and 21. This group is found in about 40-50% of patients, has fewer IgH translocations, and is thought to be associated with a better prognosis. The second subgroup is made up of non-hyperdiploid (hypodiploid) karyotypes, usually with 35-45 chromosomes, and is characterized by the loss of chromosomes 13, 14, 16, and 22. In contrast to the hyperdiploid group, the hypodiploid group has frequent IgH translocations, and is associated with a worse prognosis [2].

Recently we described a new numerical subgroup composed of hyperhaploid karyotypes with a range of 30-33 chromosomes [3]. Hyperhaploid karyotypes have most consistently been identified and recognized as a category of numerical aberrations in childhood acute lymphoblastic leukemia (ALL). In childhood ALL the hyperhaploid clones occur in two subgroups, a near-haploid group (25-29 chromosomes) and low hypodiploidy subgroup (30-39 chromosomes) and both are associated with a poor prognosis. In MM, the hyperhaploid karyotypes are characterized by a distinct set of mostly odd numbered disomies including 3, 5, 7, 9, 11, 15, 18, 19, and 21. Strikingly, this is the same set of odd-numbered chromosomes found as trisomies in hyperdiploid myeloma, with the exception of chromosome 18. This observation suggests the possibility that the origin of these different aneuploidies somehow involves the same set of chromosomes aligned on the mitotic plaque in a similar manner, but which somehow undergo a different segregation pattern due to abnormalities in the spindle apparatus and/or centrosome defects. Potentially this may be due to the same type of mitotic defect that results in the catastrophic loss of a haploid set of chromosomes in hyperhaploid ALL.

In clinical series of MM hyperhaploidy appears to be a rare numerical subgroup, or at least is rarely reported, which may be due in part to the difficulty in detecting hyperhaploid clones using current interphase FISH (iFISH) probe panels for MM. Our findings suggest that an unknown number of patients with hypotriploid or near triploid clones passed through a hyperhaploid stage without detection, and that the double chromosome number in the near triploid clones can become dominant and mask the presence of a hyperhaploid origin. Of clinical significance is the observation that the reduplication of chromosomes in the hyperhaploid clones leading to hypotriploid subclones results in these clones being masked to detection by the commonly used iFISH probes panels.

In the analysis of aneuploidy patterns in cancer by single nucleotide polymorphism arrays, it has been found that most copy number aberrations (CNAs) involve almost exactly the length of a whole chromosome or chromosome arm (arm-level) or are very short (focal) amplifications and deletions [4]. Interestingly, a higher prevalence of arm-level CNAs than focal CNAs have been found which most likely reflects major mechanistic differences in the generation of these two types of CNAs. In fact, in a typical cancer sample 25% of the genome is affected by arm-level CNAs, while only 10% show focal CNAs, with a 2% overlap. We suggest this may reflect the presence of an underlying pericentromeric instability in many cancers which promotes unbalanced arm-level rearrangements during tumor progression [5]. Recently, a computational analysis of the cancer genome databases has been used to analyze the mutational patterns in tumors. These analyses are consistent with aneuploidy patterns in cancer being the result of the accumulation of whole chromosome, arm-level, and focal CNAs [6]. Furthermore these findings are consistent with the cumulative effect of weak oncogenes or tumor suppressor genes carried on these regions of gain and loss being equal to the effect of penetrant driver genes. An underlying assumption of these analyses is that these patterns of arm-level and focal CNAs found in tumors reflect regions of gain (oncogenes), and regions of loss (tumor suppressor gene islands) [7] and that these are actively selected for and mediate tumor progression.

Interestingly, hyperhaploid clones are inherently monosomic for whole-chromosomes or chromosome arms harboring adverse cytogenetic lesions in MM including 17p, 1p, 13q, and 16q [1]. In terms of arm-level gains hyperhaploid clones demonstrate CNAs of 1q21 by jumping translocations of 1q. The 1q21-23 amplicon contains a large number of candidate oncogenes including MCL1, BCL9, CKS1B, and ANP32E among many others. The 1q21 region represents one of the most common regions of focal amplification in all cancers [4] and may therefore constitute an example of a putative oncogene island associated with tumor proliferation [6, 7].

Cytogenetic risk stratification by the International Myeloma Working Group relating to aneuploidy states that non-hyperdiploid karyotypes are considered a high-risk cytogenetic marker. We have reported the prognosis of patients with hyperhaploid karyotypes are significantly worse than patients with both hyperdiploid and hypodiploid karyotypes. Finally, when outcomes are compared in terms of the international staging system (ISS) and gene expression profiling (GEP), hyperhaploid patients have very adverse risk profiles, similar to patients with ISS stage III and GEP70 high-risk.
